# “The Very Best That It Could Be and a Lot Better Than I Would Have Imagined”: Birthing People's Experiences of Transfer From Community to Hospital

**DOI:** 10.1111/birt.12920

**Published:** 2025-05-27

**Authors:** Carrie Neerland, Arielle Skalisky, Robyn Schafer

**Affiliations:** ^1^ University of Minnesota School of Nursing Minneapolis Minnesota USA; ^2^ Division of Advanced Practice Rutgers University School of Nursing Newark New Jersey USA; ^3^ Department of Obstetrics, Gynecology, and Reproductive Sciences Rutgers Robert Wood Johnson Medical School New Brunswick New Jersey USA

## Abstract

**Background:**

Community births (those in homes or freestanding birth centers) are increasing in the US, although they still represent a small percentage of total births. Research shows that community births can offer positive outcomes for low‐risk individuals, such as fewer interventions and greater satisfaction. However, when perinatal complications arise, transfer to hospital can result in negative care outcomes and experiences. Effective integration of care and respectful communication between community and hospital providers during transfers are crucial for improving quality care measures. This study aimed to investigate the experiences and outcomes of individuals transferring from community settings to an urban US hospital with established transfer guidelines.

**Methods:**

This multi‐method study, utilizing descriptive statistics and a grounded theory approach, explores the outcomes and experiences of individuals transferring from planned home or birth center births to hospital care for intrapartum management from August 2019 to August 2020. We included participants who were 18 or older, English‐speaking, and had experienced a live birth following transfer from home or birth center to hospital. Quantitative outcomes were obtained through chart review. Qualitative interviews were conducted within 6 weeks post‐birth, recorded, transcribed, and analyzed using constant comparative analysis.

**Results:**

A total of 82 individuals transferred during the study period, with 23 participating in qualitative interviews, we identified 5 major themes: seamless transfer, teamwork, respectful care, changing expectations, and a complex relationship with autonomy and decision‐making. Participants valued smooth communication, midwife‐to‐midwife transfer of care, and the balance between autonomy and reliance on provider recommendations during transfers.

**Discussion:**

Understanding the experiences of those who transfer from community settings to hospitals is crucial for improving perinatal care. With established guidelines for transfer in place to facilitate collaboration across care providers and birth settings, transfers can be managed effectively, resulting in respectful experiences of care with positive health outcomes.

## Graphical abstract

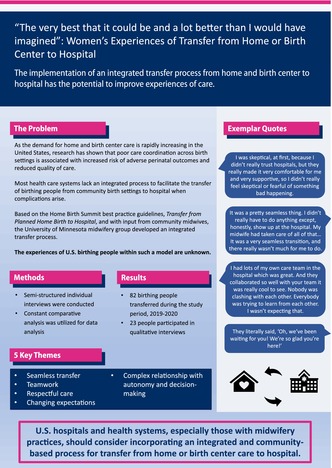

## 1 Introduction

Community births, occurring in homes and freestanding birth centers, are rising in the United States [1, 2]. Planned community births comprise a small proportion of total US births annually (2%); however, this figure has steadily increased over the past 20 years [3]. All major US perinatal organizations support the right to choose the intended place of birth [4, 5, 6, 7], though some express safety concerns [8]. Meta‐analyses and systematic reviews have demonstrated that community birth in high‐resource settings is a reasonable and safe option for low‐risk individuals and neonates associated with numerous benefits, including higher rates of spontaneous vaginal birth, fewer unnecessary interventions, and increased satisfaction and autonomy [9, 10, 11, 12, 13]. Although rates of fetal and neonatal death are higher in planned community births than in hospital births (2.4 vs. 1.2 per 1000, *p* = 0.05; 1.6 vs. 0.6 per 1000, *p* = 0.02, respectively), the absolute risk is low [14]. Integration of care and respectful collaboration between community birth settings and hospitals and health systems is essential to enhance perinatal and neonatal care quality and health outcomes [15, 16]. This includes having effective communication and timely, efficient, and reliable systems for maternal and neonatal transfer from community settings to hospitals when transfer is indicated due to changes in risk status or patient preferences [15, 16, 17, 18].

Data on transfer rates from planned US community birth to hospitals are limited. In one study based on Oregon birth certificate data, intrapartum transfer to hospitals occurred in 15.8% of planned community births (*n* = 3804). Rates of transfer were higher among nulliparous people (26.7%) than among parous individuals (8.6%) [14]. In Washington state, researchers found an intrapartum planned community birth transfer rate of 13.7% (*n* = 1455/10609), of whom most (80.4%) were nulliparous [13]. Transfers are rarely emergent; a study of 79 US birth centers reported a transfer rate of less than 2% for intrapartum emergencies [19]. Most commonly, intrapartum transfers occur due to labor dystocia [12, 20]. The study authors were unable to identify any research investigating the experiences of transfer from US community birth settings to hospitals with established transfer guidelines.

Community birth transfers are often negative experiences for birthing people, with qualitative research demonstrating delays in care, poor interprofessional communication, and mistreatment including perceived provider bias and negative judgment, blame, stigma, and even obstetric violence [21]. Inefficient and disrespectful transfers are associated with poor maternal and neonatal outcomes and negatively affect patients' and community providers' future healthcare engagement [21]. There is clear consensus on the need for integration and collaboration across birth settings and care providers to improve safety and outcomes [6, 7, 17, 22].

Recognizing the importance of developing a safe, efficient, and respectful system of community birth to hospital transfer, maternity care stakeholders convened a national Home Birth Summit in 2011 [2]. There, representatives from ethics, family medicine, midwifery, nursing, obstetrics/gynecology, public health, and other relevant disciplines collaborated to develop best practice transfer guidelines. The Home Birth Summit Best Practice Guidelines for Transfer from Home to Hospital (known as the Home Birth Summit Best Practice Guidelines) were endorsed by the American College of Nurse‐Midwives (ACNM), Midwives Alliance of North America, and National Association of Certified Professional Midwives.

These guidelines have been adapted and used throughout the United States, including Alaska, Oregon, Utah, and Washington state [23, 24, 25, 26, 27]. The Smooth Transitions project, a Washington quality improvement program, found that guidelines resulted in greater collegiality, improved communication, and increased respectful interactions among patients, midwives, and hospital personnel [23]. At the national level, the Alliance for Innovation on Maternal Health recently published a community birth transfer resource kit that includes best practices, protocols, and guidelines to ensure safe and efficient transfers from community birth settings to hospitals [28].

In 2015, the University of Minnesota hospital‐based midwifery practice developed guidelines for transfer for planned community births based on the Home Birth Summit Best Practice Guidelines. These guidelines were developed in collaboration with community midwives, obstetricians, and family practice providers and approved by representatives from hospital administration, including nursing leadership, labor and delivery medical director, and risk management. The transfer guidelines were implemented in 2015 and updated in 2018 (see Appendix S1). In addition, during this same time, midwives from across settings in this state, including midwives in this practice, were meeting to develop and enhance relationships, discuss scope of practice, and improve transfer relationships and processes.

This study aimed to explore the experiences and outcomes of people transferring from home or birth center to the hospital during late pregnancy or intrapartum in an urban, midwestern US setting with established transfer guidelines. Due to the need for foundational data, we developed a multimethod study to examine transfer experiences and outcomes. Understanding these factors is critical for improving care quality and outcomes associated with community birth transfers to inform clinical practice guidelines and health policies.

## 2 Methods

This study used a qualitatively driven multimethod concurrent design [29] to understand the experiences and outcomes of birthing people who transferred to a hospital with established transfer guidelines. Institutional review board approval was granted by the University of Minnesota.

Researchers’ positionality is as follows: The PI is a hospital‐based midwife who contributed to developing the site‐specific transfer guidelines. The second author, a midwifery student and research assistant at the hospital’s affiliated university, had no prior relationship with the study site or experience in community birth. The senior author, a hospital‐based midwife with community birth experience, had no relationship with the study site.

This study was conducted from August 2019 to August 2020 at an urban midwestern academic teaching hospital with approximately 2500 births annually. Perinatal care providers included Certified Nurse‐Midwives (CNMs), family medicine physicians, maternal‐fetal medicine physicians, nurses, and obstetric physicians. Throughout the state, there were nine birth centers staffed by CNMs and Certified Professional Midwives (CPMs) and approximately 55 licensed CPMs (email communication, Minnesota Board of Medical Practice). Home births comprised about 1.5% of all state births [3], and birth center births comprised just under 1% (email communication, Minnesota Affiliate of ACNM).

Eligible participants were age 18 or older, English‐speaking, and had experienced an antepartum or intrapartum transfer from planned community birth to hospital care, resulting in a live birth. Participants were notified about the study by the PI, student research author, or a member of the midwife team during postpartum hospital admission. Those who agreed to participate were contacted to schedule an interview and provided signed written consent. Within 6 weeks of enrollment, the PI conducted semistructured interviews in‐person, in a private setting of participants' choice (Appendix S2). Recruitment stopped when no new themes emerged from data analysis. Interviews were recorded and transcribed verbatim via a transcription service utilizing a secure platform. Deidentified transcripts were uploaded to ATLAS.ti (Version 22.0.6.0) for analysis. Health outcome data were obtained from the electronic health record.

Quantitative data were analyzed using descriptive statistics. Qualitative data were analyzed using interpretive description informed by Glaser's grounded theory method [30]. Following data immersion, the PI independently performed open coding to identify broad domains, which were then reviewed and collaboratively refined with the senior author. Axial coding by the PI further refined themes, with consensus reached through team meetings. Matrix displays visualized and summarized themes. Exemplar quotes were selected to illustrate themes and participant perspectives of the transfer experience. Bias was addressed by continuously comparing the data and ensuring that interpretations were grounded in the data, guided by theoretical sensitivity. Memoing and peer debriefing were conducted to foster reflexivity.

## 3 Results

During the study period, there were 82 transfers from planned community births to the participating hospital, either antenatally (*n* = 20, 24%) or in labor (*n* = 61, 74%). Participant demographics are reported in Table 1. Most transferred from birth centers (*n* = 67, 82%) with 14 (17%) from home birth care and 1 (1%) unknown planned site of birth. All received community birth care from either CPMs or CNMs. Most identified as White (*n* = 62, 76%), with 12 (15%) Black/African American, 3 (4%) Hispanic, 1 (1%) Asian, and 4 (5%) unknown race/ethnicity. Median maternal age was 31 (range 21–41, standard deviation [SD] 5). Most (*n* = 56, 68%) were nulliparous, with 16 (20%) primiparous and 10 (13%) multiparous.

**TABLE 1:** Maternal and newborn outcomes for transfer patients (*N* = 82).

Characteristic	*n* (%) or Mean (range)
*Primary reason for transfer*	
Pain management	30 (37)
Induction of labor	20 (24)
Hypertensive disorders	10 (12)
Augmentation of labor	8 (10)
Fetal heart rate abnormality	3 (4)
Other	3 (4)
Arrest of descent	2 (2)
Prolonged ROM	2 (2)
PPROM/PTL	2 (2)
Meconium‐stained fluid	1 (1)
Missing	1 (1)
*Mode of birth*	
Vaginal	44 (54)
Primary cesarean	31 (38)
Repeat cesarean	4 (5)
Vaginal birth after cesarean (VBAC)	3 (4)
*Gestational age (weeks)*	
37	8 (10)
38	5 (6)
39	10 (12)
40	16 (20)
41	27 (33)
42	14 (17)
43	1 (1)
Missing	1 (1)
*Newborn birth weight (g)*	
3543 (2490–4933, SD 493)	
*Newborn Apgar score*	
1‐min Apgar < 7	9 (11)
1‐min Apgar ≥ 7	73 (89)
5‐min Apgar < 7	2 (2)
5‐min Apgar ≥ 7	80 (98)
*NICU admission*	
Respiratory support	5 (6)
Triple I	2 (2)

Abbreviations: NICU, neonatal intensive care unit; PPROM, preterm premature rupture of membranes; PTL, preterm labor; ROM, rupture of membranes; Triple I, intraamniotic infection.

Twenty‐three participants (28%) completed qualitative interviews. Although individual experiences differed, several commonalities emerged in participants' narratives. Five major themes were identified: seamless transfer, teamwork, respectful care, changing expectations, and complicated relationship with autonomy and decision‐making. Each theme is described in detail below and outlined in Table 2.

**TABLE 2:** Thematic descriptions.

Themes and subthemes	Description	Exemplar quotes
Seamless transfer Subthemes: Midwife‐to‐midwife transfer; care coordination	Process of transfer was smooth and efficient with minimal burden to the patient and family	“They did a great job, it all went very well. It was super fast. We were settled in, I had an epidural, and my spouse was eating in the blink of an eye. It was like best‐case scenario” “I felt that everybody was already very prepared for me to be there. I went up to the desk, gave them my name, and then within five minutes I was in a room. Twenty minutes later I was getting pain medication” “I don't think the hospital transfer experience could have gone much better, to be honest. The people who greeted us were great, they got us in right away…I just felt like it was very seamless” “[My doula] had lots of experiences at [the hospital], she was like “It's one of the only places that you can walk in and they've seen a ton of home birth transfers. They welcome them.” I think they want to have a good relationship with the community and ensure that people feel safe coming into a hospital and not being judged. And so, we were like, ‘Great, because that is exactly our scenario.’ Everybody in our care team at home was like, ‘This is the one,’ so we trusted them, and it was a good call”
Teamwork	Care providers worked collaboratively and communicated well with other providers and patient/family	“Everybody was just so great and they collaborated so well with my team and it was just so perfect. I was just so happy to end up [where I did]” “They were really respectful, and they really respected what my doulas were saying, because my doulas really knew what they were talking about. They've been doing this for a long time. I know they are not medical professionals, but I really appreciate the fact that they respected the suggestions my doulas were giving”
Respectful care	Care was welcoming, nonjudgmental, and supportive	“They understood this wasn't my plan” “They all talked to me; they were all respectful; and they just made me feel rest assured, like everything's going to be fine”
Changing expectations Subthemes: Acknowledgment of a changing birth plan; attitudes of care providers and staff; maintaining autonomy	Expectations and perceptions were altered with hospital care that was different than anticipated	“I was trying to prep myself for my husband to advocate for me. I didn't even have to do that. I was really worried that people were going to insist on things, and it didn't feel like that at all” “I think the care team was good about still really trying to give us a choice in everything we did” “I guess my thought before I transferred was the hospital seemed a little more judgmental on transfers, or birth centers, or home births, but it did not feel like that at all when we got there. We felt very supported, and we had phenomenal care… I just feel like it was so much smoother, and more seamless, and better than I expected”
Complicated relationship with autonomy and decision‐making	Chose community birth for increased autonomy and decision‐making, however, accepting of more limited role in decision‐making and deferring to care providers	“I wanted to make my own choices and be so much more a part of my plan of care and what we did. I really liked the care. I felt that I still had some choice about my birth” “We trusted the midwives … to make good decisions for us, and they did, and it was really great” “You're happy we're here, so we can trust you. We were listening to her [the hospital midwife] and trusting her to guide us”

### 3.1 Seamless Transfer

Overall, participants found the transfer process to be smooth and efficient. This was commonly attributed to electronic record‐sharing and direct provider‐to‐provider communication, resulting in a timely and efficient intake. Participants appreciated that hospital staff anticipated their arrival and felt they were evaluated and treated promptly. Participants felt welcomed and relieved to avoid unnecessary burdens and delays in care.

It was a pretty seamless thing. I didn't really have to do anything except, honestly, show up at the hospital… They were able to get the information from my midwives, then my midwives were able to get the information from the hospital, as well, quickly. I think that it was a very seamless transition, and there really wasn't much for me to do, which was really nice. (Participant 20)

Participants felt grateful for the opportunity to transfer from community midwives to hospital‐based midwives, due to their appreciation of the midwifery model of care and its support for physiologic processes. Postpartum, several participants valued the option to return to their community‐based providers, while others elected to continue care with the hospital‐based midwifery service due to personal preference or clinical indications.

They ask me which hospital. They said, “Do you want to go to the University of Minnesota hospital or [other hospital]?” They said the University of Minnesota hospital has midwives, the other does not. I said, “OK. I want to go to the university because they have midwives.” (Participant 18)

Although participants generally experienced a seamless transition from community to hospital, some expressed concerns about financial implications and insurance coverage. Additionally, in the postpartum period, some participants' felt their expectations were unmet regarding having a clear follow‐up plans and consistent information about discharge timing

The only reason I didn't want to transfer, if I didn't have to, was just money. Just paying for the home birth and having to pay to go into the hospital. (Participant 20)

### 3.2 Teamwork

Collaboration and communication among care providers was notable in participants' narratives. They appreciated the respectful and intentional teamwork during and after transfer, valuing the inclusion of their intended care team members, such as doulas or midwives, and transparent team meetings. This team‐based approach contributed to the quality of care and increased satisfaction with the experience. Although teamwork was generally well‐received, some participants perceived the large number of unknown providers as burdensome in contrast to the few familiar providers in community‐based care.

I had lots of my own care team in the hospital which was great. And they collaborated so well with [the hospital] team it was really cool to see. Nobody was clashing with each other. Everybody was trying to learn from each other. I wasn't expecting that. (Participant 7)

### 3.3 Respectful Care

Participants recognized that hospital‐based providers showed respect for their autonomy in care decisions and provided welcoming, supportive, and compassionate care. Although some worried initially about potential judgment for choosing community birth, overwhelmingly participants felt no judgment regarding their intended birth setting and instead felt gratitude for the compassionate, humanized care they received.

They were really excellent. It seemed like they really understood that this wasn't my plan, and I think the thing that helped the most is that they said that right away. That was really nice. [Becoming emotional] I think it just took a lot of pressure off of being sad about a change. They were really great about that, and they asked if I had a birth plan, which we did, but I didn't remember any of it at that point, so they were really… “how can we make this the best experience, since this isn't what you were hoping for?” (Participant 10)

They literally said, “Oh, we've been waiting for you! We're so glad you're here!” and to have the front desk person say, “Oh, I've already made your name badges. You're already here. Great!”… to hear that, that made all the difference. (Participant 32)

Most participants reported positive interactions with the labor and birth care providers; however, some reported that pediatric providers did not fully understand their preferences for variations in the standard of care such as early discharge, delayed vaccination, or deferred newborn screening with community midwives. One participant felt negatively judged by a pediatric provider for opting to delay vaccinations, and another felt judged for requesting early discharge.

### 3.4 Changing Expectations

Participants chose home or birth center care for its unique model of care and focus on a physiologic approach to birth. However, some shared that past negative interactions with hospitals or hospital‐based providers influenced their decision not to give birth in the hospital and had low expectations for interactions with hospital staff based on these experiences. Although transfer to the hospital was unanticipated, and participants had reservations about how they would be received, they found the experience smoother and better than they had expected. Participants conveyed surprise at the prompt, high‐quality, and compassionate care that they received, which included acknowledgment of their changing birth plans, respect for their autonomy regarding care decisions, and positive attitudes of care providers and staff.

I was skeptical, at first, because I didn't really trust hospitals, but they really made it very comfortable for me and very supportive, so I didn't really feel skeptical or fearful of something bad happening. (Participant 26)

Hospital protocols or interventions did sometimes conflict with patient preferences, such as those regarding fetal monitoring and hydrotherapy. When there was a perceived lack of accurate anticipatory guidance or indication for interventions, this contributed to dissatisfaction with care.

### 3.5 Complicated Relationship With Autonomy and Decision‐Making

Autonomy and an active role in healthcare decision‐making were also important to participants who intended community birth. Although most felt their autonomy was respected, several described a complex dynamic regarding decisional autonomy. Following transfer, participants expressed a greater dependence on both community and hospital‐based providers to steer care decisions. For example, participants communicated an increased reliance on providers for decisions about pain medication and amniotomy. There were multiple rationales presented for deferring decision‐making, such as fatigue, deviations from their original birth plan, elevated risk, trust in the providers' expertise, and hospital policies. For some, knowing that their community midwife trusted the hospital midwife led to a transference of trust to the new provider.

They really made it very comfortable for me and very supportive, so I didn't really feel skeptical or fearful of something bad happening. They were just making sure I was okay. I felt like they asked for my opinion first before they made any decisions on what to do. (Participant 26)

## 4 Discussion

Although a small number of people transfer from community birth to the hospital, understanding these experiences is crucial for ensuring quality care and positive outcomes. This study found that the adoption of established guidelines and standardized processes for community birth to hospital transfer resulted in high‐quality care with smooth and effective transfers, strong provider collaboration and communication, and respectful, person‐centered care, despite changing expectations and a nuanced relationship with autonomy and decision‐making.

Quantitative data reported here were similar to findings of existing research, in that all transfers were for nonemergent reasons [19, 20], nulliparas accounted for the majority of transfers [19, 20], and the rate of cesarean birth after transfer was 43% [19]. One difference in this study was that the primary rationale for transfer was pain management (*n* = 30, 37%), rather than labor dystocia or fetal heart rate abnormalities, as reported elsewhere [19, 20].

Following the decision to transfer to the hospital, participants viewed the process as a seamless transition. Communication between community midwives and providers was perceived, overall, as smooth and nonburdensome. Establishing transfer guidelines and utilizing structured communication tools facilitate direct and timely provider‐to‐provider communication of relevant clinical information and records [31, 32]. This study supports existing research that handoff tools can improve patient care experiences and outcomes [33].

The teamwork demonstrated by the hospital and community midwives, physicians, nurses, and doulas in this study was notable to participants, who appreciated that their original care providers were respected as part of their ongoing care team. This unexpected collaboration made them feel more comfortable and well cared for. These findings reinforce existing research that team‐based care enhances care outcomes and patient satisfaction [18]. Transparency regarding the inclusion of community midwives and doulas (as desired by the birthing person), along with clear roles, frequent team meetings, and involvement of the patient and their support persons enhances feelings of respect [34].

For many participants, midwife‐to‐midwife transfer was very important. Despite changes in plans, settings, and providers, this continuity of care provided reassurance through a shared philosophy of pregnancy, labor, and birth. As noted, midwives across birth settings in this community built relationships, shared practice information, and developed a greater understanding of different scopes of practice. This collaboration has fostered trust and improved communication. When community midwives recommended transfer, they could do so confidently, knowing that support for midwifery care would be present. Hospital‐based providers should foster relationships, respect, and understanding with community midwives, and facilitate midwife‐to‐midwife transfer when possible [2, 35, 36].

Patients often had low expectations and negative perceptions of hospitals as highly medicalized, impersonal, and with judgmental staff. These expectations may stem from personal experiences, media, or a general mistrust of the medical system. However, participants in this study were surprised by the respectful and individualized care they received and found hospital‐based care to be empathetic, supportive, and professional. Respectful care should be provided in all birth settings, encompassing a nonjudgmental, shared decision‐making approach, and sufficient time to address patient questions and concerns [21].

Similar to existing research, participants chose community birth care for various reasons, including the philosophy of care in home and birth center settings, motivation for physiologic birth, nonpharmacologic pain management options, decisional autonomy, and safety [37, 38, 39]. Most felt that the autonomy experienced in the hospital surpassed their expectations, even as they often sought and trusted recommendations of hospital providers. In the dynamic labor and birth environment, factors such as fatigue, changing clinical conditions, and shifts in birth plans may lead birthing people to rely more on provider guidance, especially during transfers from planned community birth. Participants expressed trust in their community midwives and transferred that trust to hospital midwives due to the collegial and mutually respectful relationships between community and hospital midwives. Antepartal education in home and birth center care about the potential for transfer may have also contributed to this positive perception [36].

## 5 Strengths and Limitations

This study addresses the knowledge gap regarding birthing people's experiences and outcomes when transferring from community birth settings to a hospital with established transfer processes. Strengths include the timeliness of this investigation as it addresses the growing trend of community birth. Additionally, its grounded theory method allows for in‐depth exploration of individual experiences, effectively capturing the complexities and nuances of the transfer process. By highlighting the perspectives of individuals undergoing transfers, the study centers on patient experiences and needs. However, some experiences may be unrepresented, as not all individuals who transferred were interviewed. Study results may not be transferrable, as this study was conducted at a single site with collaborative provider relationships. Potential bias may exist, as the PI is a midwife at the study site; however, reflexivity, memoing, and the inclusion of researchers from diverse regions and roles mitigate bias and enhance rigor.

## 6 Implications

The findings from this study can inform clinical practice, health policy, and future research. Table 3 outlines clinical implications for improving care during transfers from community settings to hospitals, emphasizing established guidelines, direct communication between providers, and relationship‐building to enhance teamwork. This study also highlights the importance of respectful care, which includes welcoming language, honoring preferences, and educating hospital staff about community‐based midwifery. Clear communication with patients regarding transfer reasons and possible treatment recommendations is essential. As decision‐making becomes more complex with increasing levels of risk, providers should uphold patient autonomy through shared decision‐making and respect the trust between patients and their community midwives.

**TABLE 3:** Clinical implications.

Seamless transfer	Utilize Homebirth Summit Best Practice Guidelines and Model Forms—adapt to own setting, facility, or region Use direct provider to provider communication—either midwife‐to‐midwife or midwife‐to‐physician; if nonemergent but may need physician care, include all in communication
Teamwork	Develop and foster relationships among community midwives, hospital midwives, and physicians, a key step to improving collaboration and communication Demonstrate teamwork—incorporate community midwife, doula, and patient support in plan of care (as desired by the birthing person); team meetings or huddles as needed
Respectful care	Welcome respectfully: recognition that it was not their plan, use welcoming language, and offer options (for example: option not to wear gown) Honor and incorporate birth preferences as able Provide education for postpartum and newborn providers regarding community midwifery care and scope
Changing expectations	Provide clear communication with patient about reason for transfer and possible treatment recommendations Arrange for midwife‐to‐midwife transfer when possible Complete postpartum debrief with patient/family, to talk through and acknowledge experience that was different than planned
Complicated relationship with autonomy & decision‐making	Recognize the relationship and trust between the patient and community midwife Ensure shared decision‐making and patient autonomy

The study highlights the need for improved integration between community birth settings and hospitals, advocating for policies that promote seamless transfers through standardized guidelines and processes. To address financial concerns, policy reforms should improve insurance coverage for community births and transfers, reducing patient financial barriers. Comprehensive data collection on community births and transfers is essential to track trends, outcomes, and areas for improvement. Policies should support midwife‐to‐midwife transfers to maintain continuity of care, including incentives for hospitals to employ midwives and foster relationships with community midwives.

This study identifies key areas for further research. Comparative studies across regions or hospitals with varying levels of integration and collaboration can identify best practices and improvement areas. Investigating specific elements of transfer protocols that lead to positive outcomes will help refine guidelines. Additionally, examining barriers and facilitators to effective integration and respectful care during transfers can inform targeted interventions and policy changes. Finally, analyzing the economic impact of community births and transfers, including cost savings and financial burdens on patients, will provide valuable insights into their effects on the healthcare system.

## 7 Conclusion

Established transfer guidelines facilitate positive care experiences for those transferring from planned community births to hospitals. Despite initial low expectations and concerns about hospital care, participants found their transfer experiences exceeded expectations. They perceived seamless transfers, teamwork among providers, and respectful care. While participants desired autonomy in labor and birth, this often shifted during transfer, leading them to seek guidance from their community and hospital providers. With effective guidelines and collaboration, transfers from community birth settings to hospitals can be managed effectively, ensuring respectful and high‐quality, person‐centered care. These findings underscore the importance of interprofessional collaboration and the ongoing need to improve transfer processes in perinatal care.

## Conflicts of Interest

The authors declare no conflicts of interest.

## Supporting information


Appendix S1.

Appendix S2.

## Data Availability

The data that support the findings of this study are available from the corresponding author upon reasonable request.
