# Supplementary material for: “The Very Best That It Could Be and a Lot Better Than I Would Have Imagined”: Birthing People's Experiences of Transfer From Community to Hospital
Source: Birth. 2025 May 27;52(4):717–25. doi: 10.1111/birt.12920 (PMC12612382; doi:10.1111/birt.12920)
Supplement: Supplementary file 1 — Appendix S1. [file BIRT-52-717-s002.docx]

Planned Home/Birth Center Birth to M Health Transfer of Care Guidelines

*Midwife to Midwife Labor & Birth, Family Medicine Newborn Care*

**For safe facilitation of planned home/birth center birth to hospital transfer, the home/birth center provider (may be CNM or CPM) must identify emergent or non-emergent conditions (see below) and do the following:**

1. Provide information to the pregnant/birthing person prenatally and prior to labor about hospital care and procedures that may be necessary and documents a plan has been developed with the woman for hospital transfer should need arise (consistent with Minnesota Statute 147D).
2. Notify the receiving on-call M Health CNM or M Health OB/GYN MD of the incoming transfer, reason for transfer, brief relevant clinical history, planned mode of transport, and expected time of arrival at the University of Minnesota Medical Center West Bank campus.
3. Labor and delivery unit is [address of unit].
4. Continue to provide routine or urgent care en route in coordination with any emergency services personnel and address the psychosocial needs of the pregnant/birthing person during the change in birth setting.
5. Provide a verbal report, including details on current status and need for urgent care. The home/birth center provider shall also provide a copy of the prenatal record, labor record, and the documents that they are required to maintain under Minnesota Statute 147D (i.e. the written plan, informed consent, and medical consultation plan). Report to include plans for newborn care and timely screening after discharge.
6. If the pregnant/birthing person chooses, the home/birth center provider may continue to provide supportive physical and psychosocial care. The home/birth center provider can provide no medical or obstetrical care of any kind other than labor support and emotional support as may be provided under traditional doula services and only with the consent of the patient and in keeping with hospital HIPAA regulations.
7. The home/birth center provider promotes optimal communication by ensuring that the pregnant/birthing person understands the M Health CNM or MD plan of care and the hospital provider understands the pregnant/birthing person’s need for information regarding care options.
8. The home/birth center provider’s name and telephone number will be placed in the patient’s problem list in the electronic health record in order to facilitate communication and discharge follow-up planning for both birthing person and newborn.

***Clinical responsibility is transferred to the M Health CNM or M Health OB/GYN MD as deemed appropriate by CNM and MD consultation***

**The M Health Provider will:**

1. Communicate directly with the home/birth center provider to obtain clinical information in addition to information provided by the pregnant/birthing person.
2. If the pregnant/birthing person chooses, the M Health provider will accommodate the presence of the home/birth center provider as well as the pregnant/birthing person’s support person(s) during assessments and procedures.
3. The M Health CNM will consult with M Health OB/GYN MD as clinically indicated.
4. The M Health provider will coordinate with home/birth center provider to arrange follow up care for the pregnant/birthing person and newborn, and care may revert to the home/birth center provider upon discharge (per the patient’s wishes). M Health CNM will contact home/birth center provider to give discharge report.
5. Relevant medical records, such as the discharge summary, are sent to the referring home/birth center provider for discharge coordination with the appropriate patient authorization (Authorization for Release of Protected Health Information).

**Non-Emergent Conditions (M Health CNM will assume care and consult as clinically indicated):**

- Breech presentation in latent labor with reassuring fetal status
- Failure to progress in 1^st^ stage with reassuring fetal status
- Failure to progress in 2^nd^ stage with reassuring fetal status
- Maternal request for pain relief with reassuring fetal status
- Maternal Exhaustion with reassuring fetal status
- Meconium-stained amniotic fluid with reassuring fetal status
- Prolonged or premature ROM with reassuring fetal status
- Malpresentation in *latent* labor (For example, compound presentation, brow, face or other non-specified abnormal lie) with reassuring fetal status
- Pre-eclampsia w/o severe features or Gestational HTN with reassuring fetal status
- Third or Fourth degree laceration requiring repair
- Client’s desire for transfer
- Other – to be determined on admission following consultation with M Health OB/GYN MD, and with collaboration between M Health CNM and OB/GYN MD

**Emergent conditions (M Health OB/GYN MD will be consulted or assume care immediately):**

- Breech presentation in active labor
- Maternal Fever (temp >38.0 C/ >100.4 F)
- Non-reassuring fetal status (Category 2 or 3 fetal heart rate)
- Prolapsed cord or cord presentation
- Hemorrhage (Estimated Blood Loss greater than 500 ml or hemodynamic instability with HR>110 or BP <80/50)
- Malpresentation in *active* labor (For example, compound presentation, brow, face or other non-specified abnormal lie)
- Obstetric shock (Blood pressure <80/50, extremely low urine output, Fever > 38.0 or >100.4 with elevated heart rate >110 or low blood pressure <80/50)
- Suspected placental abruption, placenta previa or other abnormal intrapartum bleeding
- Suspected uterine rupture
- Retained placenta (>2 hours or active bleeding and manual removal unsuccessful)
- Uterine inversion
- Maternal seizure
- Preeclampsia with severe features
- Other – to be determined on admission following consultation with M Health OB/GYN MD, and with collaboration between M Health CNM and OB/GYN MD

**Postpartum Discharge Plan:**

Recommended discharge when key milestones are met and deemed safe for discharge:

- Clinical criteria for discharge met
- Vaccinations offered and completed
- Postpartum teaching completed
- Birth certificate and parentage paperwork completed
- Offered and attended discharge class
- Discharge medications available or prescriptions sent
- Follow-up care plan in place (home care and office visit)

**Best Practice/Quality Improvement:**

1. Both the home/birth center provider and the M Health providers will debrief case with providers and with the woman prior to discharge.
2. Review transfers with providers involved and hospital leadership (Medical and Midwifery Directors, Nurse Managers, Clinical Nurse Leaders) with shared goal of quality improvement and safety as needed and at least every six months.

***Recommendations for Newborn Care***

**The M Health Family Medicine Provider will:**

1. Assume care for the newborn after delivery and perform a complete H&P within 24 hrs of delivery (team rounding generally occurs between 7AM and 1PM daily).
2. Communicate with the mother’s M Health provider, the home/birth center provider and/or alternate outpatient pediatric provider in order to determine the best discharge plan and follow-up for the newborn.
   1. While our standard process has been well-established to prepare for discharge of healthy newborns at 24 hrs after delivery (after routine newborn screening has been completed), we are open to earlier discharge when there is a clear plan for who will assume responsibility for ensuring these recommended tests are completed (see below).

Clinical Conditions that would necessitate **48-hr observation** of the newborn after delivery

1. Untreated or inadequate treatment during labor for GBS-positive mothers during labor
2. Intra-amniotic infection during labor (infant requiring antibiotics)
3. Excessive weight loss (>10%)
4. Jaundice within the first 24 hrs
5. Signs/symptoms of neonatal abstinence syndrome or concerning maternal history or clinical exam (consistent with the Fairview Substance Use policy) warranting further evaluation and monitoring.
6. Any condition that warranted even initial admission to the NICU for stabilization and/or treatment

**Routine Discharge Criteria for Newborns**

1. Feeding well
   1. Latch score greater than 7, if breastfeeding
   2. Feeding 8-12 times per day
   3. Stable weight loss or supplementation plan, if needed
2. Afebrile, normal vital signs
3. Voiding and stooling
4. Meets GBS criteria for discharge
5. Screening tests completed
   1. Newborn metabolic blood spot screen drawn
   2. Passed pulse-oximetry screening for congenital cardiac heart disease (CCHD)
   3. Passed hearing screen or had 2 hearing attempts prior to discharge
   4. Bilirubin is either low intermediate or lower or if high intermediate or higher, needs to have 2^nd^ completed (6-12 hours) in the hospital prior to discharge
6. If desired, vaccinations completed
7. Follow up care plan in place (Home Care and office visits)
8. Car Seat trial, if indicated (infant weight <2500g; preterm)

**Early Discharge (<24 hrs) Planning**

If an early discharge (<24 hrs) is desired, and infant is medically stable, a clear plan for outpatient follow up and testing should be co-determined by the M Health provider and home/birth center provider and documented in the newborn Discharge Summary. This is to include:

1. Minnesota Department of Health (MDH) guidance for early discharge
   1. Metabolic (blood spot) screen (ideal timing 24-48 hrs)
      1. Draw blood prior to discharge regardless of age
      2. Arrange to collect a subsequent blood spot screen during the optimal time (24-48 hours of life)
   2. Hearing screen
      1. Hearing screen is valid at any age; arrange for outpatient follow up if needed
   3. Pulse oximetry screening for CCHD (ideal timing 24-48 hrs)
      1. Screening should be performed prior to discharge regardless of age
      2. Recommend that oxygen saturation levels be assessed at the first well-child check (early screening may not be accurate)
   4. **If families decline any newborn screening, MDH requires parents to sign the MDH refusal form**
2. Scheduled outpatient provider visit within 24 hours of discharge
   1. Weight check
   2. Evaluate breastfeeding
   3. Evaluate for jaundice and perform bilirubin testing (if indicated)
