# Supplementary material for: “The Very Best That It Could Be and a Lot Better Than I Would Have Imagined”: Birthing People's Experiences of Transfer From Community to Hospital
Source: Birth. 2025 May 27;52(4):717–25. doi: 10.1111/birt.12920 (PMC12612382; doi:10.1111/birt.12920)
Supplement: Supplementary file 2 — Appendix S2. [file BIRT-52-717-s001.docx]

Semi-structured Interview Questions

1. At what point (before your pregnancy or during your pregnancy), did you decide to seek care from a home birth practice/birth center practice?

2. What led you to choose home birth/birth center care?

3. At what point in your pregnancy did your midwife discuss the transfer process with you?

4. Tell me a little bit about how you and your midwife made the decision to transfer to the hospital.

5. After you were transferred to the hospital, were you cared for by a midwife or an obstetrician (ob/gyn)? Can you please describe your experience with that care team?

6. Was your home/birth center midwife able to stay with you during your labor and birth in the hospital? In what capacity?

7. Can you please tell me a little bit about your experience with the other members of your hospital care team (for example nurses, pediatricians)?

If person already discharged home:

8. After your discharge, were you cared for by your home birth/birth center midwife? Can you please say a little bit about the process of transfer back to their care?

9. Overall, how was your experience of care across the different care settings?
